# A Pilot Study of a Parent Emotion Socialization Intervention: Impact on Parent Behavior, Child Self-Regulation, and Adjustment

**DOI:** 10.3389/fpsyg.2021.730278

**Published:** 2021-10-15

**Authors:** Evalill Bølstad, Sophie S. Havighurst, Christian K. Tamnes, Egil Nygaard, Rune Flaaten Bjørk, Maria Stavrinou, Thomas Espeseth

**Affiliations:** ^1^Department of Psychology, University of Oslo, Oslo, Norway; ^2^Mindful: Centre for Training and Research in Developmental Health, Department of Psychiatry, University of Melbourne, Melbourne, VIC, Australia; ^3^PROMENTA Research Center, Department of Psychology, University of Oslo, Oslo, Norway; ^4^NORMENT, Institute of Clinical Medicine, University of Oslo, Oslo, Norway; ^5^Department of Psychiatric Research, Diakonhjemmet Hospital, Oslo, Norway; ^6^Division of Radiology and Nuclear Medicine, Department of Diagnostic Physics, Oslo University Hospital, Oslo, Norway; ^7^Department of Psychology, Bjørknes College, Oslo, Norway

**Keywords:** emotion socialization, parent intervention, self-regulation, externalizing, AX-CPT

## Abstract

Adequate emotion regulation in children is crucial for healthy development and is influenced by parent emotion socialization. The current pilot study aimed to test, for the first time in a Scandinavian population, whether an emotion-focused intervention, Tuning in to Kids (TIK), had positive effects on parent emotion-related socialization behaviors (ERSBs), and children's self-regulation, anxiety, and externalizing behavior problems. We conducted a controlled trial of the 6-week evidence-based TIK parenting program with 20 parents of preschool children aged 5–6 years and 19 wait-list controls. Assessments at baseline and 6 months after the intervention included parent-report questionnaires on parent ERSBs and child adjustment, as well as aspects of children's self-regulation assessed with two behavioral tasks, the Emotional Go/No-Go task (EGNG) and the AX-Continuous Performance Task (AX-CPT). Results showed a significant increase in reported parent emotion coaching behavior and an uncorrected significant decrease in parents' report of child externalizing problems in intervention participants compared to controls. The behavioral data showed an uncorrected significant improvement in child emotion discrimination in the control condition compared to the intervention condition, while measures of children's executive control improved from baseline to follow-up for both conditions but were not significantly different between conditions. These findings suggest that this emotion-focused parenting intervention contributed to improvement in parents' emotion coaching and their appraisal of child externalizing problems, while children's self-regulation showed mainly normative developmental improvements. Further research with a larger sample will be the next step to determine if these pilot findings are seen in an adequately powered study.

## Introduction

Starting school is a major emotional challenge for young children and adjustment during this transition is associated with school success (Margetts, 2005). Without the necessary skills in regulating emotion, cognition and behavior, and parental support to assist this, the transition can result in children displaying challenging behaviors which may inhibit their adjustment, well-being, and subsequent learning (Merrell and Tymms, 2001). How can we assist young children to develop emotional competence, and what factors may contribute to better self-regulation in preschool children?

Previous research has shown that children's skills in regulating emotions, cognitive processes and behavior, if developed early, act protectively and preventatively, reducing the risk that a child under stress will develop internalizing or externalizing problems and social difficulties (Greenberg et al., 1991; Cicchetti and Cohen, 1995; Eisenberg et al., 2001a). Self- regulation is determined by a number of processes within the child, including emotion regulation and executive functions involving cognitive and behavioral control (Posner and Rothbart, 2007; Nigg, 2017)—functions that are shaped over time by socialization processes. The child's self-regulation system is partly shaped by the quality of interpersonal interactions during early development. Parents' responses to, and coaching around, preschool children's emotional learning, termed emotion-related socialization behaviors (ERSBs), have been found to be central for children to develop self-regulation skills (Eisenberg et al., 1998; Morris et al., 2007). For example, parents' discussion of emotions with their children has been shown to be related to higher cognitive self-control in children over 1 year later, which is related to more socially acceptable child behavior (Curtis et al., 2020).

Several studies have shown that parenting programs focusing on emotion socialization that promote development of emotional competence through social and emotional interactions (Grusec, 2011), enhance both parental ERSBs and child adjustment (see i.e., Johnson et al., 2017; England-Mason and Gonzalez, 2020 for relevant reviews). However, most evaluations have only included parent-reported outcomes and no direct assessment of the child, and less is known about the impact of such programs on specific aspects of child self-regulation, including their cognitive counterparts. In their review of childhood interventions and self-control, Gagne and Nwadinobi (2018) argued for the importance of future studies to examine both cognitive and socio-emotional aspects of child development, as well as increasing the use of interventions that include a socio-emotional perspective to assist children in the important transition to starting school. Typically, interventions for child self-regulation focus predominantly on training children's inhibitory control and attention skills, with beneficial outcomes on attention, but training these specific skills may not generalize to children's functioning more broadly, such as how they cope with emotionally challenging situations in everyday life. An alternative strategy for improving children's self-regulation and adjustment is thus to work with parents to build a supportive environment that cultivates the development of self-regulation on a day-to-day basis, which may also enhance other aspects of child and family functioning. Investigating the effects of emotion socialization parenting programs on self-regulation has recently been highlighted as an important path for future studies (England-Mason and Gonzalez, 2020). Better child self-regulation and adequate parent emotion socialization are expected to reduce frequent child adjustment difficulties in preschool age children, such as anxiety and externalizing problems (Johnson et al., 2017; Robson et al., 2020).

The current study is a pilot study of an established evidence-based parenting intervention with emotion socialization from Australia, Tuning in to Kids, conducted with parents of preschool children living in Norway. The main aims of the study were to (a) investigate whether the program had an impact on parental ERSBs, (b) investigate whether the intervention had an impact on child self-regulation as measured with behavioral tasks, and (c) evaluate whether the intervention had a wider impact on child anxiety and externalizing problems. Relevant theoretical perspectives and empirical findings on interventions including parent emotion socialization, and the relation to child self-regulation and adjustment, are reviewed below.

### Child Self-Regulation and How to Aid Development of Such Skills

Regulation is a broad and comprehensive construct that involves monitoring and modulation of emotions, thoughts and behavior (Nigg, 2017). Young children's internal state or behavior can be regulated by caregivers or others, referred to as extrinsic regulation, or they can regulate themselves—called self-regulation or intrinsic regulation (Eisenberg and Spinrad, 2004; Nigg, 2017). Self-regulation includes both top-down and bottom-up processes that are mutually dependent. Top-down regulation refers to processes where children deliberately regulate their emotions, cognition or actions—including mental processes that facilitate or inhibit cognitive control and affective responses (Nigg, 2017; Thompson et al., 2020), while bottom-up processes tend to be more automated, and can be either involved in regulation or targets of regulatory processes (see Nigg, 2017, for an overview).

An important focus in the present study was how to aid development of aspects of child self-regulation that are important for school readiness, such as the ability to recognize and identify different emotions and to control impulsive reactions and chose alternative actions (Raver, 2002). Thus, self-regulation in this study was operationalized through emotion discrimination and executive control tasks. When children are able to regulate their reactions, for example by discriminating between various emotional expressions, inhibiting inappropriate responses, and showing self-control when distracted, they cope better in most situations and also elicit more supportive reactions from other people in their surroundings. Difficulty regulating negative emotions, thoughts or actions may contribute to a lack of coping in different settings, and as such linked with the development of problem behavior and maladjustment (Eisenberg et al., 2001b; Silk et al., 2003).

Further, it is important to consider possible distinctions between different self-regulation functions, which have different developmental courses, and, importantly, may be shaped by various aspects of parenting. The early development of self-regulation likely involves qualitative changes in the organizing of its components, with increasing differentiation with increased age (e.g., Akshoomoff et al., 2018; Hartung et al., 2020). This overlaps with and is followed by gradual quantitative developmental improvements, with functions developing at different speeds (Huizinga et al., 2006; Tamnes et al., 2010; Tottenham et al., 2011). Based on earlier findings, it has been suggested that parenting interventions that target a broader scope of skills including children's emotional and social development and facilitate their development across multiple domains, may be more efficient interventions than tailor-made cognitive training tasks (Diamond and Lee, 2011; Neville et al., 2013).

Even though the self-regulation system is partly shaped by the quality of interpersonal interactions during early development, few interventions have targeted parents' ERSBs as a way of influencing child self-regulation. Programs that enhance parent emotion socialization guide parents in how to directly teach children to identify and name different emotions, and help children modulate their responses in challenging situations within the context of a supportive relationship (Gottman et al., 1996). These are factors that may have both direct and indirect impact on aspects of child self-regulation, like emotion discrimination and executive control, making parent emotion socialization interventions a promising avenue for better child self-regulation. Further, a meta-analysis of the components of highly effective parenting programs found that those that included a component on emotional communication were the most effective (Kaminsky et al., 2008).

### Parent Emotion Socialization

Parent emotion socialization refer to the way parents respond to children's emotions, model emotional expressions and how well-parents regulate their own emotions (Eisenberg et al., 1998).

Gottman et al. (1996) have suggested that parent's philosophy about emotions shape their emotion socialization behaviors. Parents' attitudes, thoughts and feelings about their own and their child's emotions are related to their awareness, acceptance and coaching of specific emotions (Katz et al., 2012). Emotion coaching parents are aware of low-intensity emotions in themselves and their children, view children's negative emotions as an opportunity for intimacy and communication about emotions, validate and label emotions, and set limits and solve problems when necessary (Gottman et al., 1996). On the other hand, emotion dismissive parents tend to avoid and ignore emotions, and may convey to their children that emotional expressions are unwarranted (Gottman et al., 1996; Gottman and DeClaire, 1997; Katz et al., 2012). Eisenberg's heuristic model of parent emotion socialization outlines how parents affect children's ability to regulate emotions, cognition and behavior, and parents' ERSBs are typically operationalized as either supportive or non-supportive (Eisenberg et al., 1998; Eisenberg, 2020). Supportive parental responses correspond to emotion coaching, shape children's emotional learning and has been found to be associated with better child self-regulation and functioning (Dunn et al., 1991; Gottman et al., 1996; Lunkenheimer et al., 2007). Conversely, non-supportive or emotionally dismissive responses has been linked to deficits in children's social skills and emotion knowledge, and increased behavior problems (Lunkenheimer et al., 2007; Johnson et al., 2017). Reducing emotion dismissive responses and increasing emotion coaching parenting could therefore potentially improve children's self-regulation and prevent mental health problems during the vulnerable transition to starting school.

Currently, there are no published studies on the effects of parent emotion socialization interventions in Scandinavian countries. Most evaluations of emotion socialization interventions have so far been conducted in English speaking Western countries. Whether similar effects are seen in a Scandinavian context is not known. Norwegian parents receive substantially more economic support post-partum through the welfare system than parents in the USA, including a year of parental leave and universal access to affordable child care (Zachrisson and Dearing, 2015). These Nordic family-friendly policy schemes may contribute to strengthening attachment bonds in early childhood (Eisenberg et al., 1998; Cassidy et al., 2011). In addition, parenting in the Nordic countries is characterized as dialog-based with no tolerance for physical punishment (Hollekim et al., 2016). Thus, Norwegian parents might be more ready to learn emotion coaching and dialog-based parenting strategies, which again may contribute to enhanced child self-regulation. Norwegian parents are normally very good at taking a problem-solving approach. They would typically suggest a solution directly when exposed to the child's expression of a frustrating emotion, before using the other steps in emotion coaching. However, problem solving without responding to the emotion first can be a more dismissive response. To address this, parents in the current intervention were guided to wait with problem solving until after using the first four steps of emotion coaching when their child experiences emotions.

### Emotion Socialization Interventions and Child Self-Regulation

Parenting programs that teach emotion coaching are emerging in the evidence-based literature (Eisenberg, 2020; Havighurst et al., 2020). These teach parents' skills in noticing children's emotions, helping children understand their emotions and regulate them, assist parents to regulate their own emotions and set limits around children's behavior.

For preschool children, self-regulation is mainly assisted by the parents or other central care givers, making self-regulation a dyadic process and emphasizing the interpersonal aspects of how to manage emotions, cognitive processes and behavior (Barthel et al., 2018). By communicating accept and validate the child's expression of challenging emotions, the parent provides support and external regulation, which might help the child to practice and internalize self-regulatory skills—i.e., to inhibit and control unwanted responses, discriminate better between different emotions and to be more proactive and respond more adequate to emotional cues (Spruijt et al., 2020). In a review of research on parenting practices and child emotion regulation, Morris et al. (2017) concluded that “parents' emotional support, positive affect, emotion coaching, and use of joint strategies are all associated with more effective emotion regulation in children” (Morris et al., 2017, p. 236).

In the last two decades, studies evaluating emotion coaching interventions have been found effective in increasing children's emotional competence (see i.e., England-Mason and Gonzalez, 2020; Havighurst et al., 2020, for reviews). To directly measure child outcomes, studies have used assessment of emotion understanding, typically using Denham's (1986) puppet task, also referred to as the Affective Knowledge Test, the Emotion Skills Task, or the puppet interview (see Denham et al., 2015, for a review). In a study of an emotion coaching intervention on 75 mother-child dyads (child age 6–12) in households that had experienced intimate partner violence, Katz et al. (2020) found increased baseline vagal tone in the intervention group children, taken to index increased ability of self-soothing in stressful situations (Porges, 1995). They also found improvement in mothers' emotion regulation abilities, mother and child mental health, parent-child relationship, and mothers' confidence in dealing with child behavior problems.

While parenting has been shown to influence aspects of child emotional competence such as emotion knowledge and understanding assessed by Denham's (1986) puppet task, self-regulation processes, i.e., measured by tasks tapping executive control or emotion discrimination skills, have been less examined within the field of emotion socialization (Ferrier et al., 2014). In a review of the impact of emotion socialization parenting programs on child emotion regulation and executive functioning in preschoolers, England-Mason and Gonzalez (2020) identified three parenting programs that aimed to enhance parental emotion coaching consistent with Gottman and colleagues' definition (Gottman et al., 1996); Tuning in to Kids (TIK; Havighurst et al., 2009), Parent-Child Interaction Therapy-Emotion Development (PCIT-ED; Luby et al., 2012), and Emotion Enhanced Triple P (EETP; Salmon et al., 2014). Of the twelve studies identified, only four used observational assessment of child behavior, and of these, only one study assessed aspects of self-regulation, namely the ability to discriminate between facial expressions of emotions. In an open trial of PCIT-ED, Lenze et al. (2011) examined 8 families with depressed children aged 2–5 years, using the Penn Emotion Differentiation Test, finding that some of the children demonstrated improved emotional discrimination on the computerized task. The children also showed significant reductions in their depression symptoms after the intervention. England-Mason and Gonzalez (2020) highlight that most of the studies used operationalization of emotion regulation that tapped into broader aspects of emotional competence, making it difficult to define regulation processes properly and validly to discriminate between subdomains. As indicated above, children's ability to discriminate between different emotion expressions can help the child both to identify the emotions they observe in other individuals and contribute to decide how they should respond to other people's emotional reactions. Thus, by being more skilled with identifying emotions and modulate their response to emotional cues, i.e., as assessed by emotion discrimination and executive control tasks, children may regulate their emotional and behavioral expressions accordingly, and cope better in challenging emotional and/or relational situations. In such situations, the parents are normally the most important role models. When a parent responds to the child's emotion expression in an accepting and acknowledging way, helping their child to identity and name the emotion, this modeling and coaching parenting behavior can help the child both to identify other's expression of emotion and control their own expression of behavior. Of the few emotion socialization studies that had direct assessment of the children, none have used a population-based sample (England-Mason and Gonzalez, 2020). Thus, there is still a scarcity of studies examining the effect of emotion socialization interventions on direct assessment of child self-regulation, and especially studies that can be generalized to the wider population are needed. Further, better child self-regulation and parent ERSBs are expected to help improve child adjustment (Eisenberg et al., 2001b).

### Emotion Socialization Interventions and Child Anxiety and Externalizing Problems

Child adjustment problems are usually classified into two broad categories; internalizing problems (inward-directed problems relating to difficulties in the experience, expression and regulation of feelings, such as symptoms of depression, social withdrawal, fearfulness, and anxiety), and externalizing problems (relating to acting-out or outward-directed problems, such as problems with attention, aggression and non-compliance) (Campbell, 1995; Kovacs and Devlin, 1998). Between 7 and 12% of Norwegian preschoolers meet criteria for a mental health disorder, including internalizing or externalizing behaviors (Wichstrøm et al., 2012); indicating that prior to the transition to school, many children are already having significant problems. In preschool age, internalizing problems are mostly salient through symptoms of anxiety, rather than depression (Costello et al., 2005; Wichstrøm et al., 2012). Both internalizing and externalizing problems are often difficult to halt once they begin, they have been found to have a strong impact on later development of child psychopathology, and are also shown to often persist through to adolescence and adulthood (Trentacosta and Shaw, 2009). Interventions that target parent emotion socialization have been found effective in preventing both internalizing and externalizing problems and promoting adjustment to starting school (Salmon et al., 2009; Havighurst et al., 2010).

A recent meta-analysis of parent emotion socialization and child conduct problems found that parent practices were significantly related to both concurrent and prospective child externalizing problems (Johnson et al., 2017). Non-supportive parental behaviors predicted increasing externalizing problems, while supportive behaviors predicted a decrease in child externalizing. This is supported by a Norwegian study of the relation between parent emotion socialization and child emotion understanding and externalizing behavior, using baseline measures from the same sample as in the current study (Bjørk et al., 2020). They showed that higher levels of parental distress in response to children's negative emotion (i.e., non-supportive parenting) was associated with higher levels of externalizing child behavior and supportive parental responses were significantly correlated with child emotion understanding. In a study of an emotion socialization parenting intervention on military parents, Zhang et al. (2020) found that improvements in both parents' non-supportive behavior were associated with a decrease in child internalizing behaviors, while improvements in only mothers' non-supportive behavior were associated with a decrease in child externalizing behaviors. Further, the results showed that the intervention over 2 years had indirect effects on child behaviors through non-supportive, but not supportive, parenting. In sum, findings indicate that interventions targeting parental ERSBs contribute to reductions in child internalizing and externalizing problems and appear to work by reducing non-supportive parenting behaviors, with greatest benefits for externalizing, compared to internalizing, problems.

### The Present Study

Even though parenting programs focusing on emotional communication have shown to be among the most effective ones to enhance children's emotional competence and reduce problem behaviors (Gottman et al., 1996; Kaminski et al., 2008), interventions that teach emotion coaching are only just beginning to emerge in the literature. The present study implemented the parenting program Tuning in to Kids (TIK: Havighurst and Harley, 2010), which addresses this gap by targeting the responsiveness of parents to the emotional needs that underlie children's challenging behaviors and emotional and self-regulatory difficulties (Havighurst and Kehoe, 2021). The program aims to improve the parents' responsiveness both through their own emotion awareness and regulation and more supportive emotion coaching responses to their children's emotions. TIK, which has been found to improve preschool children's emotional competence, reduce behavior problems, and assist children as they face the transition to school (Havighurst et al., 2009, 2010, 2013; Wilson et al., 2012). Earlier studies of the TIK program with preschooler's have found that the program shows effect on both reductions in parent-reported child externalizing behavior (Havighurst et al., 2010, 2013, 2015a) and anxiety problems (Edrissi et al., 2019). Evaluations of the TIK program have been/are being conducted in the USA, China, Germany, Turkey, Iran and New Zealand (Edrissi et al., 2019; Meybodi et al., 2019; Otterpohl et al., 2020; Qiu and Shum, 2021), however, to date the program has not been tested in a Scandinavian context, to see whether it improves parental ERSBs and children's self-regulation and adjustment.

### Aims

An intervention that assists parents to learn specific skills that enhance their ERSBs is expected to mitigate children's risks related to limited emotional, cognitive and behavioral regulation. Hence, the present pilot study aimed to investigate the effects of the emotion-focused parenting program TIK in a Norwegian community setting with parents of 5–6-year-old kindergarten children just prior to the transition to school. The main goals were to examine the impact of the parenting program on parental ERSBs, child self-regulation, and child adjustment. The specific research questions tested were whether, compared to control participants, intervention: (1) parents would report increased emotion coaching and decreased emotion dismissing; (2) children would show improved self-regulation functions as measured with behavioral tasks on emotion discrimination and executive control aspects, and (3) parents would report reductions in their children's anxiety and externalizing problems.

## Materials and Methods

### Participants

Participants were parents and their children recruited from 17 kindergartens in different areas in and around Oslo. The sample consisted of 39 parents with a mean age of 41.87 years (*SD* = 4.46, range = 34–53 at T1), including 29 mothers and 10 fathers, and 40 preschool children with mean age 5.91 (*SD* = 0.32, range = 5.31–6.45 at T1), including 19 girls and 21 boys. The sample included one twin pair, where the parent delivered one questionnaire per twin. There were no differences between the intervention and control conditions in age, gender, socioeconomic measures or family status (Table 1). The children did not start school before after post-testing.

**Table 1:** Descriptive information regarding the sample, divided on intervention and control condition.

	**Intervention condition**	**Control condition**	**Difference**	
	**(*N* = 21)**	**(*N* = 19)**		
	***N* (%)/Mean (*SD*)**	***N* (%)/Mean (*SD*)**	**χ^**2**^/*t***	***p***
Child sex: Girls	10 (48%)	9 (47%)	<0.01	1.00
Child age (months)	69.8 (4.0)	71.1 (3.6)	1.21	0.29
Parents' age (years)	43.0 (5.1)	41.0 (3.7)	1.41	0.17
Parental education: four years of university or more	17 (81%)	17 (90%)	0.57	0.66
Employment	1.91	0.49		
80–100% positions	19 (91%)	19 (100%)		
**Economy**	0.82	0.52		
-Manage very well	14 (67%)	10 (53%)		
-Manage or manage well	7 (33%)	9 (47%)		
**Family status**	0.26	1.00		
-Residing partner	19 (91%)	18 (95%)		
-Single or not residing partner	2 (10%)	1 (5%)		

*All background information is at T1. Fisher's exact test was used to analyze nominal data and Student t-test for continuous data*.

### Intervention

The Norwegian version of TIK is a direct translation of the Australian TIK program (Havighurst and Harley, 2010). The program teaches parents the five steps of emotion coaching: (a) become aware of low-intensity emotions in your child, (b) view your child's emotions as a time for intimacy and teaching, (c) communicate understanding and acceptance toward your child's emotions, (d) help your child to label their emotions, and (e) if necessary, assist with choices, set limits, or problem solve [based on the five steps of emotion coaching outlined in Gottman and DeClaire (1997)]. A central aspect is to communicate that all wishes and feelings are acceptable, but some behaviors are not. In addition, the program includes activities designed to increase parents' awareness, understanding and regulation of their own and their child's emotions. This includes focusing on experiences in family of origin and exploration of attitudes toward emotions, perspective taking and empathic reflective listening skills, and promoting greater acceptance of emotions. Delivery of program content was via psycho-education, a range of exercises, group discussions, role-plays and homework activities. TIK was delivered for 2 h per week across six weekly evening sessions with two facilitators (authors Havighurst and Karevold). Norwegian translations of the original TIK parent handouts (Havighurst and Harley, 2010) were used. Fidelity checklists were completed after each session, and 100% of core program content was delivered. Each group consisted of up to 12 parents (range: 8–12).

### Procedure

Families were recruited through kindergartens. Kindergartens distributed information letters to all parents in the relevant aged preschool classes. Parents that responded with an expression of interest were then contacted by a research assistant, who gave them further information about the study and consent forms. To avoid contamination between parents in the same kindergartens, parents were allocated to either intervention or control condition based on the kindergarten they attended. Because of limited time for laboratory assessments, parents from the first 8 kindergartens that responded were assigned to the intervention, and the remaining were allocated to the control condition. Both conditions were tested at baseline. After baseline testing, those allocated to the intervention condition received TIK. Approximately 6 months after the intervention, parents in both conditions were invited to a follow-up assessment. Parents in the control condition were offered the intervention after the follow-up assessment. Children were rewarded for participation with a small gift after the testing session. Parents completed online questionnaires while the children were tested by a lab manager and 1–2 research assistants per child. The study is registered in Clinical Trials (ID: NCT04851704: https://clinicaltrials.gov/ct2/show/NCT04851704?term=NCT04851704&draw=2&rank=1), was approved by the Regional Committees for Medical and Health Research Ethics (REC: 2015/2383), and all parents gave written consent for their own as well as for their child's participation in the study. Because of ethical reasons, the material and data are not publicly shared.

### Questionnaire Measures

Questionnaires parents answered at baseline and follow-up included self-report scales about parenting, as well as child functioning. Cronbach's alpha on the scales is reported in Table 2.

**Table 2:** Cronbach alphas'of outcomes at baseline and post-intervention, and Pearson correlations between outcomes at baseline.

	**α pre**	**α post**	***n* control/Intervention**	**1**	**2**	**3**	**4**	**5**	**6**	**7**	**8**
1. PESQ emotion coaching	0.66	0.61	19/21								
2. PESQ emotion dismissive	0.82	0.81	19/21	***0.50***							
3. EGNG false alarms			17/20	−0.12	−0.31						
4. EGNG d-prime			17/20	0.03	0.28	–***0.78***					
5. AX-CPT PBI-index			19/21	0.01	0.25	0.01	0.05				
6. AX-CPT Context-d'			19/21	0.04	0.22	−0.28	**0.34**	**0.34**			
7. ECBI intensity	0.86	0.90	19/20	−0.24	−0.09	−0.13	0.09	0.08	0.11		
8. ECBI problems	0.69	0.79	19/19	−0.16	0.10	**0.34**	−0.21	−0.08	0.09	***0.62***	
9. PAS-R total anxiety	0.89	0.88	19/21	0.32	0.17	0.12	−0.10	0.05	0.04	0.28	0.26

α *Cronbach's alpha*.

*Bold = significant (p ≤ 0.05) correlation. Bold and italic = p ≤ 0.001*.

*PESQ, Parent Emotional Style Questionnaire; ECBI, Eyberg Child Behavior Inventory; PAS-R, Preschool Anxiety Scale—Revised version; AX-CPT, AX Continuous Performance Task; PBI-index, Proactive Behavioral Index; context-d'/d-prime, Hits relative to false alarms; ECBI, Eyberg Child Behavior Inventory; EGNG, Emotional Go/NoGgo task*.

#### Parent Emotional Style Questionnaire (PESQ)

The PESQ (Havighurst et al., 2010) is an adaptation of the Maternal Emotional Style Questionnaire (MESQ; Lagacé-Séguin and Coplan, 2005) and was used to measure parental beliefs about their child's sadness, anger and fear with items rated on a 5 point Likert scale. Emotion coaching beliefs were assessed with 8 items (e.g., “When my child is worried, I want to know what he/she is thinking”; “Anger is an emotion worth exploring”). Three original items related to problem solving (items 3, 12, and 15) were removed from the Emotion coaching scale, as these are interpreted as not being consistent with emotion coaching taught in the intervention (e.g., “When my child is sad, it's time to problem solve”). Emotion dismissing beliefs were assessed with 10 items (e.g., “Childhood is a happy-go-lucky time, not a time for feeling sad or angry”; “I try to change my child's worried moods into cheerful ones”). The scale was translated and back-translated by bilingual and/or fluent English-speaking developmental psychologists and research assistants.

#### The Preschool Anxiety Scale Revised (PAS-R)

The PAS-R (Edwards et al., 2010) is a 28-item questionnaire assessing anxiety and worries in children where each item is rated on a 4-point Likert scale. The PAS-R consists of five subscales of anxiety equivalent to the DSM-IV classifications: separation anxiety, social anxiety, physical injury fears, obsessive compulsive, and generalized anxiety. The PAS-R was designed as an adjunct to clinical interview diagnosis for screening those children at-risk for anxiety problems and provides an indication of the child's levels of anxiety. Good construct validity of the PAS-R has been established earlier (Edwards et al., 2010; Wang and Zhao, 2015).

#### The Eyberg Child Behavior Inventory (ECBI)

The ECBI (Eyberg and Robinson, 1983) is a 36-item parent-report questionnaire that measures children's behavior problems. The inventory has two subscales: an Intensity score, assessing frequency of behavior problems (e.g., “Physically fights with sisters and brothers”) with ratings on a 7 point Likert scale, and a Problem score, measuring whether the rater believes the behavior to be a problem or not. The questionnaire has good psychometric properties (Burns and Patterson, 2000; Axberg et al., 2008), and has been widely used and validated (Reedtz et al., 2008; Reedtz and Martinussen, 2011).

### Direct Assessment of Child Behavior

#### Emotional Go/Nogo Task (EGNG)

The EGNG task used in the current study (Hare et al., 2008) was an adaptation from the Go/NoGo task—a well-established cognitive paradigm. When modified with emotional stimuli in the form of faces with different positive and negative emotional expressions serving as either target or non-target, the task allows for behavioral assessment of emotion discrimination, emotion regulation and cognitive control, which are related, yet separable processes (Tottenham et al., 2011). Neuroimaging studies, including studies with children, have been used to validate that the task can dissociate activity in prefrontal top-down control systems from activity in subcortical limbic regions for both negative and positive emotions (Hare et al., 2008; Somerville et al., 2011). The participants were presented with pictures with different facial expressions that was displayed on a screen. When a named target expression was presented, the subjects were instructed to press a button as fast as they could (Go trials), while the subjects were asked to withhold pressing if the facial expression was different from the named target expression (NoGo trials). To ensure that the predominant reaction of the subjects was to respond, the target expression/Go trials occurred in a majority of the trials. Participants were not told what the facial expressions in NoGo trials was, but just instructed to withhold pressing a button for all facial expressions that was not the Go expression. The task consisted of four blocks of different pairs of facial expressions (sad-neutral, neutral-sad, angry-neutral, and neutral-angry), where either the emotional or the neutral expression served as a Go or NoGo stimulus (e.g., when neutral was the Go stimulus, the emotional expression served as the NoGo stimulus). Each block included 30 trials, of which 20 were Go trials and 10 were NoGo trials. Each picture was shown for 1,200 ms and interstimulus interval varied randomly in the range 1,250–1,750 ms. Nine practice trials were administered to ensure that participants understood the task and could execute the responses. Three children did not complete enough trials at baseline, and two children did not complete at post testing. Stimulus was presented and responses recorded using the E-Prime 2.0 software (Psychology Software Tools, Pittsburgh, PA). For the current study we used d-prime, a sensitivity index which balances both number of hits and number of false alarms, as a measure of emotion discrimination (specifically the ability to tell specific emotional faces apart); and false alarms across all blocks as a measure of self-regulation in the context of both emotional and neutral stimuli.

#### AX Continuous Performance Task (AX-CPT)

The AX-CPT task (a version of the classic Continuous Performance Test, Rosvold et al., 1956) is among the tasks most frequently used to study adaptive cognitive control by cognitive and clinical neuroscientists (Cohen and Servan-Schreiber, 1992; Servan-Schreiber et al., 1996). In particular, the task makes it possible to distinguish between proactive or reactive control modes (Braver et al., 2007; Braver, 2012). Whereas, proactive control refers to anticipatory and sustained maintenance of goal representations (i.e., the context), reactive control reflects transient stimulus-driven reactivation of goal representations. Each trial of the standard AX-CPT consists of two displays: first, a contextual cue is presented, then, after a delay, the probe is presented, and participants decide whether the probe is a target or not and respond by pressing the appropriate button. We adapted the standard AX-CPT to use with children. The AX-CPT have been shown to validly index proactive and reactive control modes in children in preschool age (Chatham et al., 2009; Chevalier et al., 2015). In our “Angry bird” version of the AX-CPT, a green pig or a blue bird were used as contextual cues, and a red apple, or purple grapes were used as probes. The combination pig—apple was defined as the target and constituted 70% of the trial combinations. Children were told that the pig likes apples, but not grapes. When the pig is displayed, followed by the apple, they should press a right-hand key. The remaining combinations constituted 10% each. If the grapes follow the pig, they should press a left-hand key. The blue bird does not like anything, and they should therefore press the left-hand key regardless of the identity of the second picture. Left and right key presses were counterbalanced between participants. A practice block of ten trials was presented to familiarize the children with the task. The instructions given were to respond as quickly and accurately as possible.

The trial started with a 700 ms fixation period. The cue stimulus followed (pig or bird) was presented for 500 ms, followed by a fixation cross for 1,500 ms. Then the probe stimulus was presented (apple or grapes) for another 500 ms followed by a 1,500 ms screen with a fixation cross. In this interval the children had to press the response button. A feedback screen with a smiley figure lasting 500 ms followed if the child responded correctly. A final fixation cross of 500 ms duration followed and this ended the trial. There were 120 trials in total, divided into 4 experimental blocks of 30 trials. The children were encouraged to complete all trials, but were told that they could terminate whenever they felt too tired to continue. In session 1, 36 participants completed 120 trials or more (one participant completed 180 trials). The remaining three participants completed 30, 60, and 86 trials, respectively. In session 2, 37 participants completed 120 trials. Data from one participant is missing, and the remaining two completed 30 and 60 trials, respectively. Sensitivity analyses, where children with <120 trials on AX-CPT are excluded, find similar effects on AX-CPT as presented in Table 3. E-prime 2.0 (Psychology Software Tools) was used for stimulus presentation and recording of responses.

**Table 3:** Descriptive information of outcomes, condition differences at baseline and effects of intervention.

	**Pre**		**Post**		**Effects**				
	**Intervention**	**Control**	**Intervention**	**Control**	**Condition differences at baseline**	Intervention effects (Time^*^Condition)			
	**(*n* = 21)**	**(*n* = 19)**	**(*n* = 21)**	**(*n* = 19)**						
	**Mean (*SD*)**	**Mean (*SD*)**	**Mean (*SD*)**	**Mean (*SD*)**	**Estimate (CI)**	***p***	**Estimate (CI)**	***p***	**Cohens *d* (CI)**
**Parental ERSBs**									
PESQ emotion coaching	31.0 (3.6)	32.7 (2.6)	33.3 (3.5)	32.1 (2.5)	−1.8 (−3.8. 0.2)	0.08	**3.0 (1.0, 5.0)**	0.004^*^	**0.75 (0.30, 1.20)**
PESQ emotion dismissive	27.2 (5.5)	26.4 (5.9)	29.9 (4.8)	28.0 (6.3)	0.7 (−2.8, 4.3)	0.68	1.2 (−1.5, 3.9)	0.39	0.27 (−0.17, 0.71)
**Child self-regulation**									
EGNG false alarms	9.5 (5.7)^a^	8.4 (7.4)^d^	9.1 (5.2)	8.1 (6.5)^d^	1.0 (−3.1, 5.0)	0.64	0.1 (−3.8, 4.0)	0.95	0.02 (−0.44, 0.48)
EGNG d-prime	1.8 (0.5)^a^	1.7 (0.8)^d^	1.9 (0.6)	2.4 (1.1)^d^	0.1 (−0.4, 0.6)	0.69	–**0.6 (**–**1,2**, –**0.1)**	**0.02**	–**1,1 (**–**1.61**, –**0.62)**
AX-CPT PBI-index	0.2 (0.3)	0.0 (0.3)	0.3 (0.4)	0.2 (0.4)^c^	0.2 (−0.0, 0.4)	0.09	−0.1 (−0.5, 0.2)	0.53	−0.22 (−0.66, 0.23)
AX-CPT context d-prime	1.2 (1.4)	0.7 (1.5)	2.5 (1.0)	2.2 (1.4)^c^	0.5 (−0.3, 1.4)	0.21	−0.2 (−1.0, 0.7)	0.69	−0.15 (−0.60, 0.29)
**Child externalizing and internalizing**									
ECBI intensity	117.5 (18.1)^a^	105.0 (18.4)	110.4 (20.2)	102.9 (23.8)	11.7 (−1.2, 24.6)	0.08	−4.2 (−13.4, 4.9)	0.36	−0.42 (−0.87, 0.03)
ECBI problems	7.3 (4.4)^b^	5.5 (2.9)	5.0 (3.8)^b^	5.0 (4.1)^c^	1.8 (−0.7, 4.3)	0.15	–**2.2 (**–**4.3**, –**0.1)**	**0.05**	–**0.47 (**–**0.94, 0.00)**
PAS-R total anxiety	33.5 (15.8)	31.5 (14.9)	32.4 (15.3)	30.9 (11.8)^c^	2.0 (−7.3, 11.3)	0.67	0.3 (−6.3, 6.8)	0.94	0.05 (−0.40, 0.49)

*Fixed effects from mixed models without covariates. All results are based on sum scores. There are no significant differences between intervention and control condition at baseline, neither in any of the outcomes nor in child gender, parental age or parental education. No confounders are included in the analyses because of this similarity between conditions at baseline and because of the limited number of participants. EGNG, Emotional Go/NoGo task; d-prime, Hits relative to false alarms; AX-CPT, AX Continuous Performance Task; PBI-index, Proactive Behavioral Index; PESQ, Parent Emotional Style Questionnaire; ECBI, Eyberg Child Behavior Inventory; PAS-R, Preschool Anxiety Scale – Revised version*.

a *n = 20*,

b *n = 19*,

c *n = 18*,

d *n = 17*.

*Bold = significant (p ≤ 0.05) effect of intervention*.

* *Significant (p ≤ 0.05) after adjusting for multiple (n = 11) tests as suggested by Hochberg and Benjamini (1990)*.

Accuracy was estimated for each trial type (AX, AY, BX, BY) in all experiments. The proactive behavioral index (PBI) was computed for each participant by relating AY and BX error rates, as in previous publications (e.g., Braver et al., 2009), according to the following formula:


$$\begin{array}{l} PI=\frac{E_{AY}-E_{BX}}{E_{AY}+E_{BX}} \end{array}$$

Where E, the error rate for each condition, is computed using the following formula which avoids complications when the number of errors is small or zero:


$$\begin{array}{l} E=\frac{number\;of\;errors+0.5}{\;number\;oftrials+1} \end{array}$$

The PBI varies between −1 and +1: the closer the score is to +1, the more proactive-like is the strategy of the participant; the closer the score is to −1, the more reactive-like is the strategy. A score of 0 means equal amount of AY and BX errors.

Context-d' was calculated for each participant based on AX hit rates and BX false-alarms. This measure is derived from the d' of signal detection theory and indicates sensitivity to distinguish the different types of probes (target X in AX trials vs. non-target X in BX trials) (Stanislaw and Todorov, 1999). Larger values of context-d' indicate greater sensitivity. In the current study, we used both PBI and context-d' as a measure for the executive control aspect of self-regulation, the former control strategy, and the latter control performance.

### Statistical Methods and Preliminary Analyses

Univariate linear mixed effects models were used to investigate condition differences in changes over time. There were no significant differences between intervention and control condition at baseline on any of the outcome (Table 3) or background variables (Table 1). No confounders or random effects were included in the mixed models due to this similarity between conditions and because of a limited number of participants. The model included the fixed effects of time, condition and the interaction effect between time and condition, in which the interaction effect is the estimate of the intervention effect. In addition, the effect size is presented as Cohen's d calculated based on the mean change in each condition, pre-intervention standard deviations and correlation across both conditions between pre- and post-test results (Morris and DeShon, 2002) using the online calculator no 4 at https://www.psychometrica.de/effect_size.html#repeated. As each outcome represent results regarding different outcomes, we present *p*-values and effect sizes from univariate analyses. In addition to uncorrected *p*-values, *p*-values adjusted for multiple analyses as suggested by Hochberg and Benjamini (1990) and calculated in R version 3.4.4 are also reported. We used IBM SPSS statistics version 25 for all other analyses, significance levels were set at 0.05, and all tests were two-tailed.

Assumptions of homoscedasticity and normally distributed residuals, and thus linearity, were investigated by visual interpretation of Predicted Probability (P-P) plots and scatterplots of predicted values and residuals, and were fulfilled for all models. The univariate analyses did not include any confounder variables; thus multicollinearity was not tested. However, correlation between outcomes, in addition to internal consistency within each outcome, are presented in Table 2.

There were few missing cases on each individual item within each scale (mean 1.6%). The participants either reported on almost all items per measure (maximum two missing) or did not report on a large proportion of the items. Thus, we only calculated scores for participants with less than three missing items per scale, based on mean imputation method within each measure. Because of partly overlapping information with Table 3, information on changes over time for intervention and control condition is placed in Supplementary Table 1. Correlations between changes in the outcome variables are presented in in Supplementary Table 2.

## Results

### Descriptive Analyses

Cronbach's alpha and correlations between the study variables are reported in Table 2, while means and standard deviations for the intervention and control conditions are reported in Table 3.

### Parental ERSBs

There was a significant interaction between time and condition, indicating that the intervention condition had greater increases in their self-reported emotion coaching abilities [d (95% CI) = 0.30, 1.20] compared to the control condition (Table 3). There were no significant differences between the conditions in terms of changes in emotional dismissiveness (Table 3).

Regarding changes over time, the intervention condition, but not the control condition, had significant increases in both parenting skills measured by PESQ, i.e., emotion coaching and dismissiveness, from T1 to T2 (see Supplementary Table 1).

### Child Self-Regulation

There was no significant intervention effect on the children's results on the continuous performance test (AX-CPT), nor on the number of false alarms on the EGNG task (Table 3). However, the control condition showed an uncorrected significant increase in d-prime on the EGNG relative to the intervention condition [*d* (95% CI) = −1.61, −0.62].

Regarding changes over time for each condition, the children in the intervention condition did not have significantly different results at T1 vs. T2 on the EGNG measures or on the PBI from the AX-CPT task. However, both conditions improved their context-d' from T1 to T2 on the AX-CPT task, indicating greater sensitivity for the probes. The only significant change over time for the EGNG measures was improvement on the d-prime for the children in the control condition, indicating better emotion discrimination. Further details regarding changes over time for each condition are presented in Supplementary Table 1.

### Child Anxiety and Externalizing Problems

Regarding intervention effects, there was an uncorrected significantly larger decrease in parent-reported child behavioral problems on the ECBI in the intervention condition than in the control condition [*d* (95% CI) = −0.94, 0.00] (Table 3). There were no significant differences in change over time between the conditions for intensity of behavior problems or for child anxiety on the PAS-R, even though there was a medium effect size for intensity of behavior problems (*d* = 0.42) (Table 3).

Regarding changes over time, the number of child behaviors reported by their parents as a problem decreased significantly across time for both conditions, whereas intensity of behavior problems and total anxiety did not change significantly (Supplementary Table 1).

## Discussion

This pilot study aimed to test the impact of the TIK parenting program on parental ERSBs, child self-regulation and adjustment in Norway, using both parent-reported and behavioral measures. TIK is an emotion-focused parenting program that has established evidence in community and clinical samples in Australia. By using an intervention-control group design, this study sought to test—for the first time—whether the program would hold promise in a Scandinavian setting. The main results showed that reported emotion coaching parenting behavior in the intervention condition had increased significantly 6 months after the intervention, while there was no increase in the control condition over time. In addition, the parents in the intervention condition reported a significant larger decrease in how problematic they perceived their child's externalizing behavior, compared to the control condition. Finally, the direct measures of child behavior showed an uncorrected significant improvement in emotion discrimination in the control condition relative to the intervention condition, but no significant condition differences for measures more specifically targeting self-regulation. The results will be discussed more thoroughly below.

### Impact of TIK on Parental ERSBs

As hypothesized, results showed that the parents who received the intervention reported significantly higher levels of emotion coaching at 6-month follow-up, while control parents did not report any increase. The size of the increase was *d* = 0.75, thus a medium to large effect size, indicating that the intervention was effective in a Scandinavian setting—as the main goal of the TIK program is to enhance parents' emotion coaching (Havighurst and Harley, 2010). The increase in emotion coaching is consistent with earlier findings from Australia on the impact of TIK in preschoolers, both with a community trial (Havighurst et al., 2009, 2010) and with a selected sample of TIK with fathers (Wilson et al., 2016). In contrast to another recent pilot on the TIK study in Iran (Aghaie Meybodi et al., 2017), we found support for the hypothesis that the TIK intervention would lead to increased emotion coaching. Removing the three items on problem solving might have contributed to a more valid measure of emotion coaching. However, while several other TIK studies have found that the main changes were reductions in parent emotion dismissiveness (Havighurst et al., 2009; Wilson et al., 2012, 2016; Aghaie Meybodi et al., 2017), the current study found no significant differences in parents' emotional dismissiveness between the conditions. Further, change in emotion dismissiveness across both groups was related to change in emotion coaching, but not to any of the other outcomes. A possible reason is that an increase in knowledge regarding what is positive parental behavior inflate reports of both parental outcomes. The lack of relations between the increase in dismissiveness and changes in the child, neither parental reported or tested, may be because the increase in dismissiveness is rather a change of their evaluation of their own behavior than a change in what they are actually doing. However, the sample from the present pilot study is small and predominantly well-educated, thus, these findings need to be replicated in a larger more diverse sample to make any reliable conclusions.

### Impact of TIK on Child Self-Regulation

The current study used two behavioral assessment paradigms as indication of child self-regulation; an emotion discrimination task (EGNG) and an executive control task (AX-CPT). In the emotion discrimination task, the results indicated that children in the control condition showed increased accuracy on emotion discrimination over time, pointing to age relevant improvements in the control condition for this aspect of self-regulation. This is consistent with other studies showing improvement in accuracy on EGNG with age (Lewis et al., 2006; Tottenham et al., 2011). However, another study did not find any accuracy changes in EGNG with age (Cohen Kadosh et al., 2014), supporting the lack of changes in the intervention condition in the present study. The executive control task included measures of the children's use of control strategies and control performance, and the results indicated that children from both conditions improved their performance sensitivity—pointing to better self-control in task performance. This result is generally in line with previous studies which have compared AX-CPT performance in preschool children with children aged 6–10 years (Chatham et al., 2009; Lucenet and Blaye, 2014; Chevalier et al., 2015), suggesting that the new version of AX-CPT adequately tapped the expected developmental changes over time in the executive control aspect in children's self-regulation (Diamond, 2013). However, this conclusion should be interpreted with caution since our longitudinal design does not allow for distinguishing between developmental effects from mere training effects.

In contrast to our hypothesis, there was no significant intervention effect for children on either of the self-regulation tasks. The only significant effect when the groups were compared was improvement on the emotion discrimination task for the children in the control group, thus opposite to our hypothesis of improved self-regulation for the children in the intervention group. Few, if any, studies have examined these relations between an emotion socialization intervention and preschoolers' emotion discrimination and executive functions before, thus, we compare the findings with other intervention studies using similar measures of child self-regulation. In a study that aimed to improve parent-child interaction through educating parents in how to support and scaffold the development of cognitive, social-emotional and self-regulatory skills in children that promotes adaptive behavior, they analyzed results from 70 children and their parents, who were randomly assigned to either intervention or control condition (Spruijt et al., 2020). The intervention consisted of four group meetings with home assignments in between. The parents showed improvements in supportive presence and less intrusiveness after the intervention, but they did not find that the educational condition led to improvement in child functioning after a 6-month follow-up. They did however find enhanced attentional control and executive functioning, as measured with the Amsterdam Neurological Tasks (including the Go/No-Go paradigm), in the four- to eight-years-old children of those parents that had improved after the program (Spruijt et al., 2020). The authors argued that most of the studies that have found gains in self-regulatory skills, have been on samples with larger initial deficits or low-income families in studies with longer follow-up (i.e., Diamond and Lee, 2011; Neville et al., 2013; Diamond and Ling, 2016), such that 6 months might be too short follow-up to find detectable effects in a well-functioning sample. These arguments are relevant for the present study, as our sample is economically well off and highly educated, and there was a short time period between the parent intervention and the assessment of child regulation. As suggested and shown by Ferrier et al. (2014), observed emotion expression and executive functioning in preschoolers will over time influence each other. Impact from parent ERSBs may take longer to manifest in the development of children's self-regulation.

Interestingly, in the current study the control condition increased their accuracy of hit vs. false rate significantly more than the intervention condition on the EGNG, indicating that they improved more with emotion discrimination over time. Several non-significant tendencies in the data point to slightly poorer functioning children in the intervention condition compared to the control condition (see pre and post means on child indicators in Table 3), which may explain why only the control condition showed a learning effect of the test. As mentioned in the Methods, parents in the intervention conditions were the first to make contact and show their interest in participating in the study. All though speculative, an explanation could be that the intervention parents struggled more with their children's behavior, making them more motivated to participate, thus contributing to a slight bias in condition assignment with the intervention children starting off with somewhat poorer self-regulation. This could also point to the reason for null-findings in self-regulation for the intervention condition participants, i.e., that the intervention children were more challenging to influence compared to the control condition, and also why only the latter seemed to gain more self-control over time. Future studies should test the relationship between parent emotion socialization and child self-regulation behavior in a larger sample with randomized groups and a longer follow-up period.

### Impact of TIK on Child Anxiety and Externalizing Behavior

Consistent with the hypothesis, the findings showed a significantly larger decrease in parental reports of child externalizing behaviors in the intervention condition compared to the control condition. We found, however, that the parents differed only on their report of the externalizing behaviors as a problem, and not on intensity levels. Still, as the present study is a pilot study with a small sample size and the intervention is offered to parents and only indirectly addresses child behavior, this is quite a strong indicator. Different versions of TIK have been shown to reduce externalizing problems (i.e., Havighurst et al., 2004, 2015a,b; Duncombe et al., 2014; Lauw et al., 2014), and of these, one was with a large study conducted with preschool children from a population-based sample (Havighurst et al., 2010). Regarding smaller samples like the present pilot study, TIK has been shown to have an impact on externalizing problems in preschool children in the pilot study of Aghaie Meybodi et al. (2017). They, however, used a selected sample only including children with behavior problems, thus a sample expected to have a greater potential for change. Interestingly, our results are different to findings on emotion socialization interventions in general, where most of the studies have shown that a decrease in non-supportive behavior has a stronger effect on reducing child externalizing, compared to an increase in supportive parenting (see Johnson et al., 2017 for a meta-review) that we found in our study. Future research with larger samples is needed to investigate whether this difference in the impact of supportive vs. non-supportive parenting is especially relevant for externalizing behaviors in a Scandinavian culture.

Contrary to our hypothesis, we did not find a significant intervention effect with the parental reports of children's anxiety. This is consistent with findings from earlier studies of TIK with preschool children (Aghaie Meybodi et al., 2017), which also failed to show significant differences between intervention and control conditions on child anxiety. Only one study using the TIK intervention has so far shown a reduction in parent reported child anxiety symptoms (Edrissi et al., 2019), however this pilot study used a selected sample including children with increased levels of anxiety symptoms. Our finding is also in contrast to Zhang et al. (2020), who found that a parent emotion socialization intervention contributed to lower child internalizing 2 years later. This result was with families that were combat deployed, with expected higher risk for child problems, and the study had 1.5 year longer follow-up than the current study. The impact of emotion socialization interventions on child anxiety problems is still relatively unclear, and future studies should explore more closely what factors in the intervention contribute to improved child internalizing symptoms.

Overall, there are indications that TIK seems better suited for preventing externalizing problems rather than anxiety problems in children within the normal symptom range. Internalizing problems in children are by definition harder to identify for parents, and such symptoms may not be identified and addressed at all, whether with supportive or non-supportive parenting behaviors. Thus, it might be that topics included in the TIK intervention, like showing empathy for (visible) emotion expressions and helping children to label emotions, are easier to apply for parents of children struggling with externalizing behavior problems.

Finally, the current findings are based on a pilot study which had the statistical power to detect medium to large, but not small effects. The functions of a pilot study, like the present with a small sample, can test if changes occur consistently with the theory; whether the measures detect effects and perform as expected; whether the intervention holds promise and is acceptable in the target population. In that respect, findings from the present study were consistent with an emotion socialization perspective and TIK's theoretical background, showing significant changes in parental ERSBs and child behavior in the expected directions and supporting the adaptation of assessment for child self-regulation.

## Limitations

The results of the present study should be considered in light of the following limitations. First, the sample was small, reducing the chance of detecting true effects, but also increasing the effect size variability due to sampling error. Second, the study outcomes were dependent on parent reports for child adjustment, and on self-reports for parental ERSBs. This is measurement which is vulnerable for i.e., social desirability bias, thus multi-informant measures such as including teacher reports of child adjustment should be considered in future studies. Third, the intervention condition included the parents from the first eight kindergartens that showed interest in participating the study, which may have contributed to a selection bias. The parents were put in groups depending on their address and which group time points that suited them, independent of the kindergartens they belonged to, but it could still be that the intervention condition included more motivated parents. The level of parent-reported externalizing problems in the intervention condition tended to be higher, nearly significantly different at baseline (*p* = 0.08), compared to the control condition. This may be an indication of a selection bias, in that the parents who perceived their children to have more externalizing problems, where most motivated and then quick to reply to the study invitation. Kindergarten-level differences may potentially impact the results. This was not measured in the current study; thus, future studies should include such information. It was limited associations between child self-regulation and child adjustment, which may indicate that how children perform on a computer task might be quite different from how they behave in challenging situations during the day. Finally, the parents in the sample were predominantly well-educated, Caucasian Europeans, and middle class. The findings, therefore, may not be generalized to other populations. Further, to be able to detect any effects from a *parent* intervention to direct assessment of *child* functioning, a longer follow-up than 6 months may be crucial.

## Conclusions

In spite of the abovementioned limitations, results from the present pilot study found a medium to large effect of the TIK intervention on parental ERSBs, supporting the effectiveness of the TIK program. Parents in the intervention condition showed significant increases in parent emotion coaching compared to parents in the control condition, and they reported their child's externalizing behavior as significantly less problematic after the intervention. Even with a relatively small sample, the TIK program show significant impact on parental ERSBs and parent reported child externalizing behavior. However, the study did not show any intervention effects on direct assessment of child self-regulation, but only on improvements in the intervention group based on reports from parents. To be able to detect effects from an emotion socialization intervention directed toward parents, to direct assessment of child self-regulation, it is recommended that future studies include larger samples and measure the effects over a longer follow-up than 6 months.

## Data Availability Statement

The raw data supporting the conclusions of this article will be made available by the authors, without undue reservation.

## Ethics Statement

The studies involving human participants were reviewed and approved by The Regional Committees for Medical and Health Research Ethics (REC: 2015/2383). Written informed consent to participate in this study was provided by the participants' legal guardian/next of kin.

## Author Contributions

EB: conceptualization, methodology, formal analysis, investigation, data curation, project administration, supervision, funding acquisition, writing—original draft, writing—review and editing. SH: conceptualization, methodology, investigation, validation, resources, writing—review and editing. EN: methodology, formal analysis, writing—review and editing. CT and TE: conceptualization, methodology, investigation, resources, software, formal analysis, writing—review and editing. RB: methodology, investigation, writing—review and editing. MS: methodology, investigation, formal analysis, writing—review and editing. All authors contributed to the article and approved the submitted version.

## Funding

EB and MS were supported by internal funding from the Department of Psychology, University of Oslo, Norway. CT was supported by the Research Council of Norway (#288083, #223273). TE was supported by the Research Council of Norway (#231286/F20).

## Conflict of Interest

SH wishes to declare a conflict of interest in that she may benefit from positive reports of the Tuning in to Kids program. The remaining authors declare that the research was conducted in the absence of any commercial or financial relationships that could be construed as a potential conflict of interest.

## Publisher's Note

All claims expressed in this article are solely those of the authors and do not necessarily represent those of their affiliated organizations, or those of the publisher, the editors and the reviewers. Any product that may be evaluated in this article, or claim that may be made by its manufacturer, is not guaranteed or endorsed by the publisher.
