# Supplementary material for: A Pilot Study of a Parent Emotion Socialization Intervention: Impact on Parent Behavior, Child Self-Regulation, and Adjustment
Source: Front Psychol. 2021 Oct 15;12:730278. doi: 10.3389/fpsyg.2021.730278 (PMC8554311; doi:10.3389/fpsyg.2021.730278)
Supplement: Supplementary file 1 [file Data_Sheet_1.PDF]

Appendix

Supplemental Table 1. Changes over time for intervention and control condition

	Intervention condition		Control condition		Effects of time			
	Pre (*n* = 21)	Post (*n* = 21)	Pre (*n* = 19)	Post (*n* = 19)	Intervention condition		Control condition	
	Mean (*SD*)	Mean (*SD*)	Mean (*SD*)	Mean (*SD*)	Estimate (CI)	*p*	Estimate (CI)	*p*
**Parenting:**								
PESQ Emotion Coaching	31.0 (3.6)	33.3 (3.5)	32.7 (2.6)	32.1 (2.5)	**2.4 (1.0, 3.7)**	**.001***	-0.6 (-2.1, 0.8)	.38
PESQ Emotion Dismissive	27.2 (5.5)	29.9 (4.8)	26.4 (5.9)	28.0 (6.3)	**2.7 (0.9, 4.6)**	**.005***	1.6 (-0.4, 3.5)	.12
**Child outcomes**								
EGNG False alarms	9.5 (5.7)[a]	9.1 (5.2)	8.4 (7.4)[d]	8.1 (6.5)[d]	-0.4 (-3.0, 2.2)	.74	-0.5 (-3.4, 2.3)	.70
EGNG d-prime	1.8 (0.5)[a]	1.9 (0.6)	1.7 (0.8)[d]	2.4 (1.1)[d]	0.1 (-0.3, 0.4)	.69	**0.7 (0.3, 1.1)**	**.001***
AX-CPT PBI-index	0.2 (0.3)	0.3 (0.4)	0.0 (0.3)	0.2 (0.4)[c]	0.1 (-0.1, 0.4)	.30	0.2 (-0.0, 0.5)	.07
AX-CPT context-d’	1.2 (1.4)	2.5 (1.0)	0.7 (1.5)	2.2 (1.4)[c]	**1.3 (0.7, 1.8)**	**< .001***	**1.4 (0.8, 2.0)**	**< .001***
**Parent reported child outcomes**								
ECBI Intensity	117.5 (18.1)[a]	110.4 (20.2)	105.0 (18.4)	102.9 (23.8)	-6.3 (-12.7, 0.1)	.05	-2.1 (-8.6, 4.5)	.52
ECBI Problems	7.3 (4.4)[b]	5.0 (3.8)[b]	5.5 (2.9)	5.0 (4.1)[c]	**-2.5 (-4.0, -1.0)**	**.002***	-0.3 (-1.9, 1.2)	.65
PAS-R Anxiety	33.5 (15.8)	32.4 (15.3)	31.5 (14.9)	30.9 (11.8)[c]	-1.1 (-5.6, 3.4)	.62	-1.4 (-6.2, 3.4)	.57

Notes. Fixed effects from mixed models. The models had time, condition and interaction between time and condition as fixed effects. The models included no covariates or random effects. All results are based on sum scores. All models were run twice, to entangle the effect of time of both conditions.

[a] n = 20, [B] n = 19, [c] n = 18.

Supplemental Table 2. Pearson correlations between changes in outcomes divided by group

	n control\ intervention	1.	2.	3.	4.	5.	6.	7.	8.	9.
1. PESQ Emotion Coaching	19 \ 21		.35	.43	**-.62**	.30	.35	.07	.44	.12
2. PESQ Emotion Dismissive	19 \ 21	.24		.05	-.09	-.08	.01	.10	.13	.12
3. EGNG False alarms	16 \ 20	.04	.13		**-.63**	.26	.04	.08	.43	.19
4. EGNG d-prime	16 \ 20	**-.51**	-.30	**-.64**		-.43	-.22	-.12	-.39	-.08
5. AX-CPT PBI-index	18 \ 21	**-.62**	.21	-.09	**.50**		**.59**	-.05	**.51**	-.02
6. AX-CPT Context-d’	18 \ 21	-.38	.01	-.26	.48	**.61**		.14	.29	.03
7. ECBI Intensity	19 \ 20	-.04	-.24	.11	.21	.01	.02		.30	***.70***
8. ECBI Problems	18 \ 18	.14	-.05	-.13	-.12	-.28	-.17	**.53**		.46
9. PAS-R Total anxiety	18 \ 21	-.07	.00	.22	-.14	.24	.39	-.29	-.39	

Notes. Pearson correlations for control group at bottom left and for intervention group at top right side of table. Bold = significant ($p \leq .05$) correlation. Bold and italic = $p \leq .001$. PESQ = Parent Emotional Style Questionnaire; ECBI = Eyberg Child Behavior Inventory; PAS-R: Preschool Anxiety Scale – Revised version; AX-CPT = AX Continuous Performance Task; PBI-index = Proactive Behavioral Index; context-d’/d-prime = Hits relative to false alarms; ECBI = Eyberg Child Behavior Inventory; EGNG = Emotional Go/NoGgo task.
